# Idiopathic Mesenteric Phlebosclerosis Secondary to Chinese Herbal Medicine Intake in an Older Adult

**DOI:** 10.4269/ajtmh.22-0772

**Published:** 2023-09-11

**Authors:** Akira Kawashima, Akira Shimomura, Takeshi Inagaki

**Affiliations:** Department of General Internal Medicine, National Center for Global Health and Medicine, Tokyo, Japan

## CLINICAL IMAGE

An 84-year-old woman with anemia and a 4-week history of discomfort on the right-side of the abdomen received treatment for hypertension, dyslipidemia, and osteoporosis. She had been treated with the traditional Chinese drug Kami-shoyo-san (Tsumura & Co., Tokyo, Japan) for several years for neuropsychiatric symptoms secondary to menopause. Physical examination revealed a soft abdomen with no tenderness.

**Figure 1.**
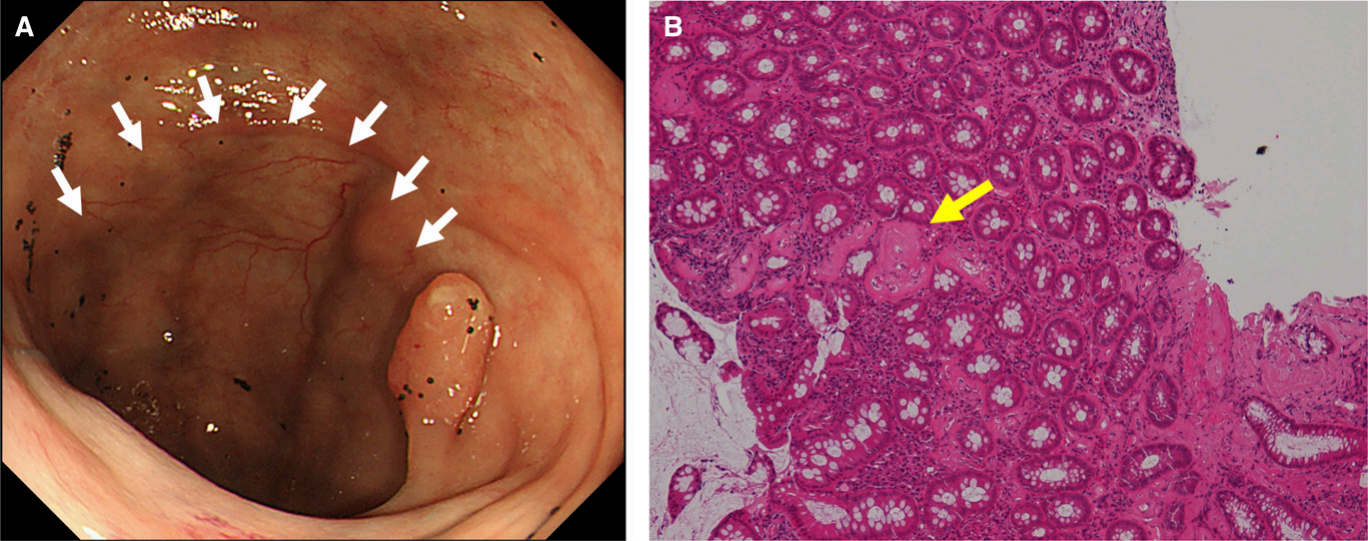
(**A**) Colonoscopy revealing bluish, edematous mucosa and decreased blood vessel permeability in the ascending colon (white arrows). (**B**) Hematoxylin and eosin staining of a biopsy specimen from bluish mucosa showing unstructured deposits in the interstitium and vessel wall (yellow arrow) (magnification 200×).

**Figure 2.**
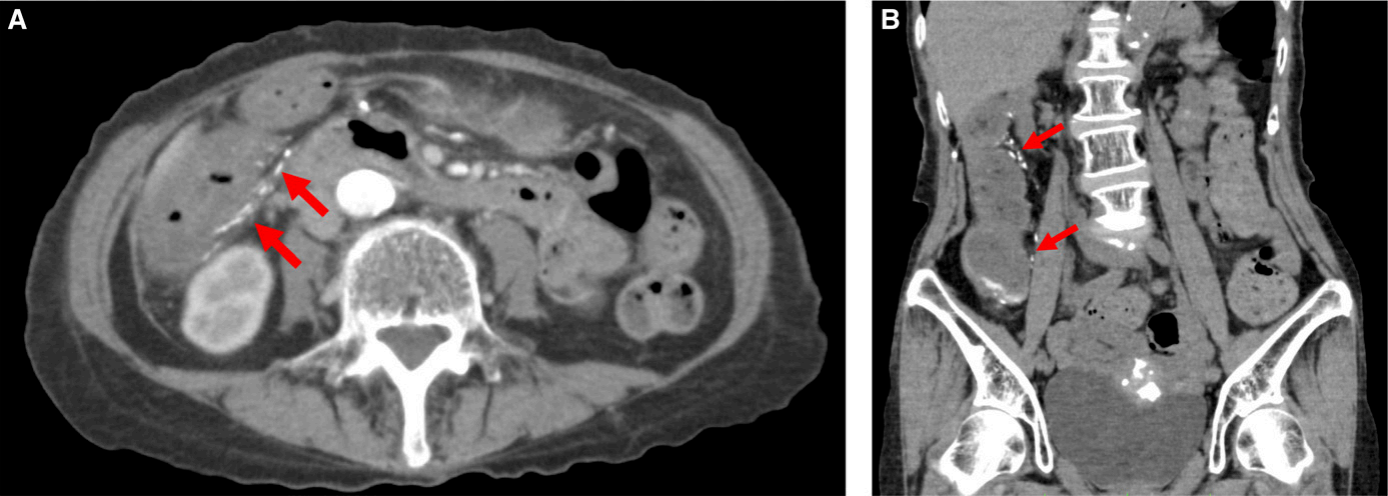
(**A**) Axial view and (**B**) coronal view of the abdominal computed tomography scan showing colonic wall thickening with calcification of the superior mesenteric vein in the ascending colon (red arrows in **A** and **B**).

A colonoscopy revealed bluish edematous mucosa, decreased blood vessel permeability, and multiple small ulcers with white slough extending from the cecum to the hepatic flexure (Figure 1A, white arrows). Biopsies of the ulcer and bluish mucosa revealed necrotic deposits of the lamina propria, submucosal layer of the ascending colon, and small vessel wall on hematoxylin and eosin staining (Figure 1B) but were negative for amyloid deposits on Congo red staining. Contrast-enhanced computed tomography (CT) showed edematous thickening of the ascending colon and multiple linear calcifications distributed on the superior mesenteric veins (Figure 2, red arrows).

Based on the typical combination of endoscopic and radiological findings, the patient was diagnosed with idiopathic mesenteric phlebosclerosis (IMP); herbal drug treatment was immediately discontinued, conservative treatment was initiated, and regular endoscopic and radiological observations were scheduled.

Idiopathic mesenteric phlebosclerosis is a rare disease reported mostly in East Asian countries (including Japan). Herbal medicines containing geniposide (called “Sanshishi” in Japanese) are one of the major causes of IMP.^1^ Drinking Chinese medicinal liquor as well as herbal medicines was shown to be a risk for IMP.^2^ Clinical manifestations of IMP include right-sided abdominal pain and abdominal distention. In the literature, CT has revealed colon wall thickening and calcification along the superior mesenteric vein.^3^ A colonoscopy showed edematous mucosa, dark purple discoloration, erosions, and ulcerations of the right-sided colon due to chronic ischemic changes.^3^

When considering IMP as a differential diagnosis, clinicians should recall typical findings on endoscopic and CT images. It is useful to assess the severity and extent of IMP, such as the involved bowel segment, to indicate conservative treatment outcomes and surgical needs.^4^ Because there are no approved treatment guidelines for IMP and no specific therapeutic agents, discontinuation of herbal consumption is the first priority, and the prognosis for IMP is good when diagnosed early.^5^

Most of the IMPs in the literature review were reported in Japan, China, and Korea, but two were reported in Canada and one each in Germany, the United States, and the United Kingdom; in addition, clinicians should be cautious about oral medications in travelers from Asia.^5^
